# Editorial: Sexual Interaction in Digital Contexts: Opportunities and Risks for Sexual Health

**DOI:** 10.3389/fpsyg.2022.872445

**Published:** 2022-03-15

**Authors:** Nicola Döring, Nicole Krämer, Matthias Brand, Tillmann H. C. Krüger, Johanna M. F. van Oosten, Gerhard Vowe

**Affiliations:** ^1^Media Psychology and Media Design, Institute of Media and Communication Science, Department of Economic Sciences and Media, Technische Universität Ilmenau, Ilmenau, Germany; ^2^Social Psychology: Media and Communication, Department of Computer Science and Applied Cognitive Science, Faculty of Engineering, University of Duisburg-Essen, Duisburg, Germany; ^3^General Psychology: Cognition and Center for Behavioral Addiction Research (CeBAR), Department of Computer Science and Applied Cognitive Science, Faculty of Engineering, University of Duisburg-Essen, Duisburg, Germany; ^4^Clinical Psychology and Sexual Medicine, Department of Psychiatry, Social Psychiatry and Psychotherapy, Hannover Medical School and Center for Systems Neuroscience, Hannover, Germany; ^5^Youth and Media Entertainment, Faculty of Social and Behavioral Sciences, University of Amsterdam, Amsterdam, Netherlands; ^6^Communication and Media Studies, Center for Advanced Internet Studies (CAIS), Bochum, Germany

**Keywords:** internet sexuality, cybersex, online sexual activities (OSA), sexting, pornography, voice assistants, sex robots, sexual consent

Sexual intercourse initiated *through* a sex dating online platform, sexually suggestive interpersonal communication *via* a messaging app, or flirtation *with* a voice assistant are examples of sexual interaction in digital contexts. In all of these cases the interaction is linked to both sexual arousal and digital technology. The digitalization of human sexuality has been an issue of polarized public and academic debates for decades. Concerns about negative health outcomes (e.g., online pornography addiction and online sexual abuse) go hand in hand with hopes for positive health effects (e.g., improved sexual intimacy and wellbeing).

There have been prior special issues on forms of digitalized sexuality that we partly build on and partly strive to advance: Delmonico and Griffin (2012) in their special issue focus almost exclusively on the “dark side” of cybersex, namely clinical and criminal aspects. Sevcikova and Daneback (2014) cover both positive and negative effects and also address consent and commerce—but only with a view to Internet technology. Twist and McArthur (2017) encourage therapists to explore both the challenging and beneficial roles that technologies play in their patients' sexual lives. Ngo et al. (2017) edited a special issue on the legal regulation of sexting, while Potenza (2018) edited a special issue on the personal and public health aspects of online pornography and its problematic use. A recent special issue edited by Dibble and McDaniel (2021) addresses the romantic dimension of online dating and a special issue on “current and emerging aspects of cybersexuality” edited by Shaughnessy (2022) is under preparation.

In this Frontiers Research Topic, we aimed to explore recent developments in the field of sexual interaction in digital contexts by describing their contexts and characteristics as well as acknowledging their opportunities and risks for sexual and overall wellbeing. In order to advance the field, our special issue follows the systematization proposed by Döring et al. and distinguishes sexual interaction *through, via*, and *with* digital technology. Theoretical and empirical articles are included, namely qualitative and quantitative studies with cross-sectional, longitudinal and experimental designs. Data from Australia, Canada, Germany, the Netherlands, and the United States are presented.

In the first article, Döring et al. provide a conceptual analysis and explain the new concept SIDC (Sexual Interaction in Digital Contexts) with its three types: sexual interaction *through, via*, and *with* digital technology. For a comprehensive understanding and analysis of each of the three types of SIDC, four key causes and consequences as well as the two main mediators (consent and commerce) are suggested and elaborated.

## Sexual Interaction *Through* Digital Technology

In the second article, Ponseti et al. address one main subtype of sexual interaction *through* digital technology, namely searching for new sexual and romantic partners and initiating sexual encounters through online dating platforms and apps. Based on 13 earlier studies, the selective review argues that women and men in the modern digital dating arena act according to ancient sex-typical strategies and evolutionary programs. They also explain why women appear to be more successful than men in reaching their sexual goals in online dating.

The sexual face-to-face communication and interaction of some couples is shaped *through* their joint use of pornography. Earlier research has shown that joint use can inspire open communication and sexual exploration within couples. The third article by Kohut et al.—based on four separate survey studies—confirms that couples who watch pornography together also report higher sexual and relationship quality. However, when one partner uses pornography alone and the other partner does not use pornography at all, couples report lower sexual and relationship satisfaction.

## Sexual Interaction *Via* Digital Technology

Flirtatious and openly sexual interpersonal communication takes place more and more often *via* digital media. People engage in sexy self-presentation on their social media profiles and exchange nude selfies or suggestive text messages via smartphone or computer. Although this behavior can strengthen social relationships, it also comes with risks. The fourth article by Reer et al. reports about a longitudinal study among more than 1,000 German internet users aged 14–64 years. They found that online sexual engagement is fairly widespread and predicts future online sexual victimization.

In the fifth article, Courtice et al. differentiate the gender and relationship contexts in which non-consensual sexting or—more broadly—non-consensual TMSI (technology-mediated sexual interactions) occur. Based on an online survey of about 450 university students in Canada, it is shown that young women tend to receive more undesired sexual content from known non-partners and strangers than men. Young men, on the other hand, send non-consensual sexts to strangers more often than women.

The sixth study by Budde et al. is focused on an even younger demographic group, namely adolescents aged 16–17 years and their sexting experiences. In group discussions with about 20 girls and 20 boys, three types of adolescent sexters were identified: the “experimenters” who approve of sexting as a way of experimenting with sexuality, the “reflexive-criticals” who question social norms around sexting, and the “disapprovers” who reject and abstain from digital sexual practices. This qualitative study gives young people a voice and demonstrates the different perspectives on sexting among adolescents.

Based on a representative national survey of about 21,000 Dutch 12–24-year-olds, the seventh article by Boer et al. reveals that 4% of the participants have shared someone else's sext in the last 6 months. Being male, aged 12–14 years, engaging in frequent social media usage, watching online porn, having sexual experience, and being subjected to sext-sharing themselves is associated most strongly with sext-sharing. The article calls for the integration of sexting and non-consensual sext-sharing as critical topics in both sex education and media literacy programs.

## Sexual Interaction *With* Digital Technology

Sexual interactions *with* digital media such as online erotica or pornography can be very engaging. Although some individuals and couples enjoy their interactions with sexually explicit digital media content and report mostly neutral or positive effects, others develop problematic (e.g., addiction-like) use patterns. In the eighth article, Markert et al. report on a neuroscientific experiment comparing healthy men's brain reactions to pornographic images while being in a negative vs. a neutral mood. It turned out that negative mood alone was not enough to trigger increased reaction to pornographic cues. Only men who had a higher solitary sexual motivation reacted more strongly to pornography when they were in a bad mood.

Although traditional digital pornography elicits interaction in the form of media selection and para-social interaction with porn performers, some digital platforms provide sexually explicit content and invite users to actively interact with it in the form of liking, rating or commenting. So-called “slutpages,” for example, provide the opportunity to upload sexual photos of third parties and have them rated by the community. The ninth article by Clancy et al.—based on an online survey of a convenience sample of more than 1,100 young adults from the United States and Australia—links interactions on “slutpages” with online image-based evaluative voyeurism (OIBEV). Men and women are curious about the sexual content, but women are 3–4 times more likely than men to check if their own photos are being published.

In the age of upcoming artificial sexual partners with interactive capacities such as software bots or hardware robots, it is important to better understand underlying mechanisms. The tenth study by Szczuka investigates effects of sexual suggestive and flirtatious verbal interactions with a human being vs. a voice assistant based on the Sexual Interaction Illusion Model (SIIM). An online experiment with more than 250 participants revealed that the voice assistant evoked more interest in further messages and in the technology itself, while the human was still perceived to be more sexually attractive and flirtatious.

We hope that the Frontiers Research Topic on Sexual Interaction in Digital Contexts will inspire future studies and global collaborations in this growing, interdisciplinary field.

## Author Contributions

ND, NK, MB, TK, JO, and GV contributed to planning the Research Topic. ND, NK, MB, TK, and JO served as associate editors. ND took the lead in writing and editing the editorial. All authors contributed to the editorial and approved the submitted version.

## Funding

This research was supported by the DFG (German Science Foundation, Bonn, Germany) by means of two discussion forums (KR 2240/9-1) in preparation for a special priority program. In addition, the CAIS (Center for Advanced Internet Studies, Bochum, Germany) funded a research meeting of the authors.

## Conflict of Interest

The authors declare that the research was conducted in the absence of any commercial or financial relationships that could be construed as a potential conflict of interest.

## Publisher's Note

All claims expressed in this article are solely those of the authors and do not necessarily represent those of their affiliated organizations, or those of the publisher, the editors and the reviewers. Any product that may be evaluated in this article, or claim that may be made by its manufacturer, is not guaranteed or endorsed by the publisher.
